# California poppy (*Eschscholzia californica*), the Papaveraceae golden girl model organism for evodevo and specialized metabolism

**DOI:** 10.3389/fpls.2023.1084358

**Published:** 2023-03-02

**Authors:** Annette Becker, Yasuyuki Yamada, Fumihiko Sato

**Affiliations:** ^1^ Plant Development Lab, Institute of Botany, Hustus-Liebig-University, Giessen, Germany; ^2^ Laboratory of Medicinal Cell Biology, Kobe Pharmaceutical University, Kobe, Japan; ^3^ Graduate School of Biostudies, Kyoto University, Kyoto, Japan; ^4^ Bioorganic Research Institute, Suntory Foundation for Life Science, Kyoto, Japan; ^5^ Graduate School of Science, Osaka Metropolitan University, Sakai, Japan

**Keywords:** flower development, benzylisoquinoline alkaloid, Ranunculales, evo devo, VIGS (virus-induced gene silencing)

## Abstract

California poppy or golden poppy (*Eschscholzia californica*) is the iconic state flower of California, with native ranges from Northern California to Southwestern Mexico. It grows well as an ornamental plant in Mediterranean climates, but it might be invasive in many parts of the world. California poppy was also highly prized by Native Americans for its medicinal value, mainly due to its various specialized metabolites, especially benzylisoquinoline alkaloids (BIAs). As a member of the Ranunculales, the sister lineage of core eudicots it occupies an interesting phylogenetic position. California poppy has a short-lived life cycle but can be maintained as a perennial. It has a comparatively simple floral and vegetative morphology. Several genetic resources, including options for genetic manipulation and a draft genome sequence have been established already with many more to come. Efficient cell and tissue culture protocols are established to study secondary metabolite biosynthesis and its regulation. Here, we review the use of California poppy as a model organism for plant genetics, with particular emphasis on the evolution of development and BIA biosynthesis. In the future, California poppy may serve as a model organism to combine two formerly separated lines of research: the regulation of morphogenesis and the regulation of secondary metabolism. This can provide insights into how these two integral aspects of plant biology interact with each other.

## 1 Phylogeny, biogeography and growth conditions

**Figure 1:**
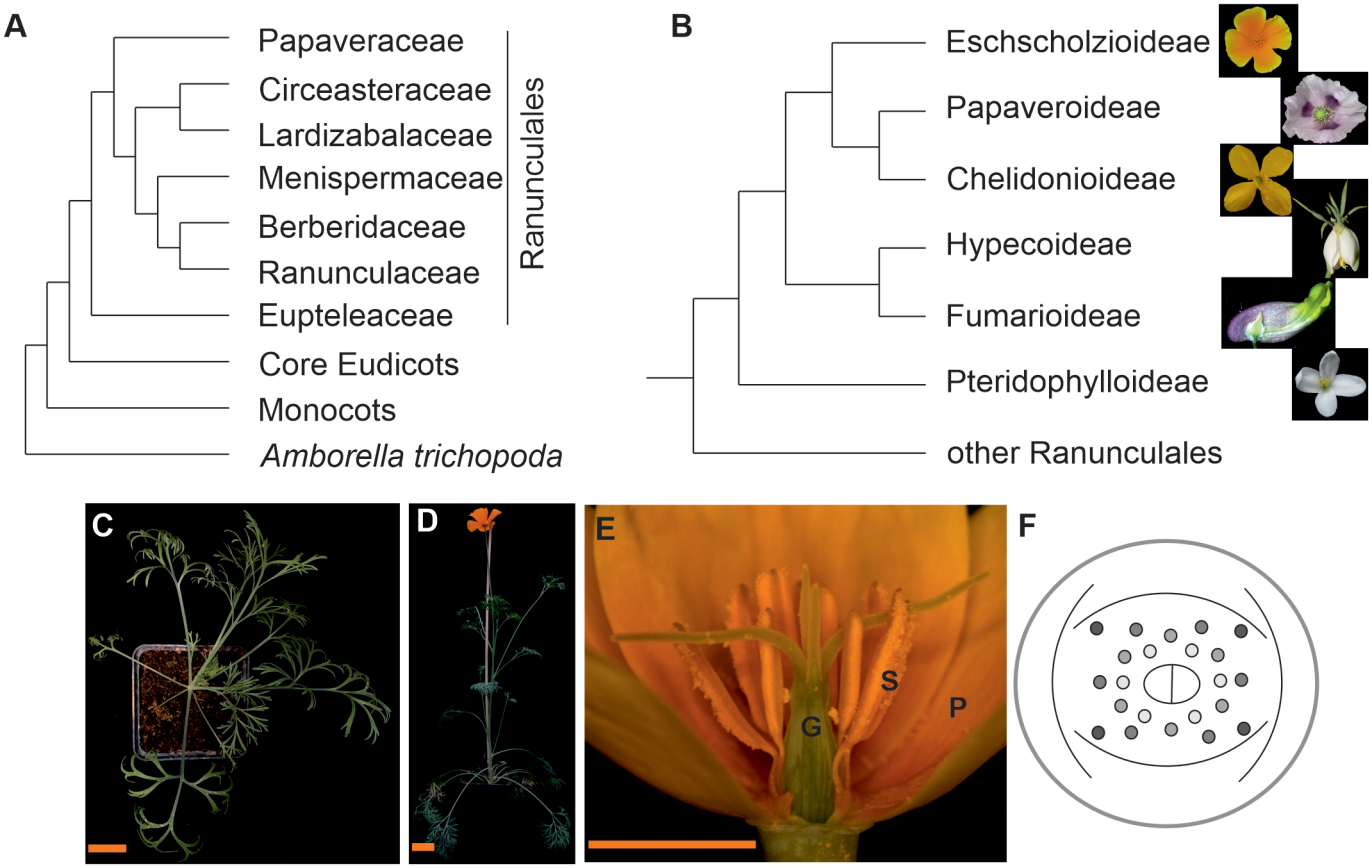
Simplified angiosperm phylogenies focusing on the phylogenetic position and morphology of California poppy. **(A)** Phylogeny of the Ranunculales [according to Lane et al. (2018) and Hoot et al. (2015)], **(B)** phylogeny of the Papaveraceae s.l. on subfamily level based on Hoot et al. (2015). Fotos next to the branches show subfamily representatives, from top to bottom California poppy*, Papaver somniferum, Chelidonium majus*, *Hypecoum leptocarpum*, *Capnoides sempervirens, Pteridophyllum racemosum.* **(C)** top view on California poppy in its vegetative phase, **(D)** side view of a flowering plant, **(E)** side view of a California poppy flower (G, gynoecium; P, petal; S, stamens) and **(F)** floral diagram showing the sepal cap, two whorls of petals, several whorls of stamens and a bicarpellate gynoecium (photos are from Dominik Lotz and Doudou Kong, Gießen, Germany, and Natalia Pabon-Mora, Medellin, Colombia). The size bar in C and D correspond to 3 cm, the size bar in E is 1 cm.

*Eschscholzia californica* is a member of the Papaveraceae family of the order of Ranunculales, which is sister to the core eudicots (**Figure 1A**, Hoot et al., 2015; Lane et al., 2018). Sister to all Papaveraceae is the enigmatic *Pteridophyllum racemosum*, with fern-like leaves and bell-like white flowers in a loose inflorescence. *Eschscholzia californica*, with common name California poppy, Golden poppy, or Cup of Gold, belongs to the subfamily of Eschscholzioideae, which is the sister group to both, the Papaveroideae (including *Papaver somniferum*, opium poppy) and the Chelidonioideae (**Figure 1B**).

California poppy is growing as an annual or perennial and is native to western North America. Its native range covers the Columbia River in Northern Oregon to Baja California, a peninsula separating the Gulf of California from the Pacific Ocean. It is found from the Pacific Coast to the Great Basin, including the Sierra Nevada and the Mojave Desert (Cook, 1962). In the past 200 years, California poppy was introduced by man to Chile, South Africa, Australia including Tasmania, and New Zealand, where they became naturalized weeds (Cook, 1962; Leger and Rice, 2007). For the Chilean invasive population, the population history was documented in astonishing detail: California poppy was introduced to the Chilean coastal cities as an ornamental plant during the 1890s and spread from there along railway tracks. Within less than 100 years, the species spread 240 km south and 520 km north of their original points of introduction, including all of the Mediterranean climate region of Chile in its range. Furthermore, California poppy habitat covers an enormous altitudinal range between 0 and 2000 m.a.s.l. in Chile (Véliz et al., 2012) with traits differing between coastal and high altitude plants: coastal plants were shorter, required more time to flower, and produced fewer flowers and fewer seeds per fruit, regardless if the plants were collected from California or Chile. After being introduced to Chile, California poppy has improved its abilities to colonize disturbed environments and evolved robust patterns of local adaptations, all in only 150 years (Leger and Rice, 2007).

Owing to its invasive nature and Mediterranean climate natural range, California poppy is easy to grow and a commonly grown ornamental plant. The plants (Becker et al., 2005; Wege et al., 2007), require little space, 42 plants/m² can be grown comfortably, but up to 90 plants/m² are possible, and standard greenhouse conditions (6 cm pots, standard potting mix, 21°C – 25°C day, 15°C-18°C night, natural light supplemented with fluorescent tubes or mercury lamps, 16 h light/8 h darkness). California poppy does not require governmental permits to be raised and does not require CITES documentation as it is not an endangered plant species.

## 2 California poppy morphology

Cotyledons of California poppy are deeply lobed, and the shoot apical meristem (SAM) gives rise to highly dissected, silvery green leaves forming a rosette. Once the shoot elongates, the SAM converts into an inflorescence meristem, which continues to release leaves. When the uppermost two leaves are formed, the inflorescence meristem converts into a terminal flower meristem. In the leaf axils of the uppermost cauline leaves, new secondary meristems may form. California poppy leaf size and shape depends on the position and age of the leaves: length and degree of dissections increases with age during the vegetative phase, but decreases after transitioning to flowering (Becker et al., 2005).

California poppy has large flowers with four brightly colored petals. The flowers across natural populations are diverse, with petal color in the northern and coastal habitats of its native range being more yellow and the central and southern populations show a larger portion of orange petal color. Stamen numbers range between 16 and 39 with the southern population developing fewer stamens normally (Cook, 1962). California poppy flowers are composed of two sepals fused into a cap-like structure that dehisces when the petals fully elongate. Four petals are 1-3 cm in length and width and are arranged in two concentric whorls (Becker et al., 2005). The petals are not only intensely colored by carotenoids but also have a silky appearance. This effect is caused by a thick, prism-like ridge that runs along enormously elongated petal cells and focuses light to the pigments at the abaxial epidermis cell side (Wilts et al., 2018).

Further inside the flower, the stamens form, with four stamens in the first stamen whorl and all other consecutive whorls forming six stamens. The stamens are composed of a short filament and long, bilobed anthers. The basal part of the anthers is often dark brown with all other parts colored bright yellow. In the center of the flower, the gynoecium is composed of two fused carpels forming a long, superior ovary topped by a short style. Four stigmatic protrusions are covered with papillae that often bend down towards the floral base. A floral tube forms that surrounds the ovary, which is adorned with a torus rim to which the sepals and petals are attached. In the ovary, two rows of ovules are attached to the two placentae (Becker et al., 2005).

After fertilization, the gynoecium develops into a slender capsule of around 5 cm in length at maturity. The capsule dries out and the valves separate from bottom to top from each other releasing the seeds explosively, scattering them up to 1.5 m (Cook, 1962; Becker et al., 2005).

### 2.1 Molecular regulation of vegetative and reproductive development, examples from functional studies in California poppy

Several studies examining the role of California poppy orthologs of *Arabidopsis thaliana* developmental regulators in recent years contributed to our understanding of gene function conservation. The focus of these studies was on floral development, with an emphasis on carpel development. The advantage of California poppy in these studies is its comparatively simple floral and fruit morphology allowing direct comparisons with long established model species like *Arabidopsis*.

Highly conserved developmental regulators, such as MADS-box transcription factors from California poppy show partially similar functions to *Arabidopsis*, such as specification of floral organ identity. However, their regulation differs from that of *Arabidopsis* and a higher number of genes in the poppy allows for sub- and possibly neofunctionalization. For example, the *AGAMOUS* (*AG*) orthologs of California poppy are required for stamen and carpel organ identity, and the *APETALA3* (*AP3*) and *PISTILLATA (PI)* orthologs for petal and stamen organ identity (Yellina et al., 2010; Lange et al., 2013). The two *AG* orthologs and three *AP3* orthologs are differentially expressed in the flower. *EScaAG1* shows a stronger expression, especially in carpels and fruits. *EScaAG2* is expressed mainly in stamens and only very little in other floral tissues. This divergence in expression suggests that *EScaAG1* is more important for carpel identify and possibly also for the regulation of floral meristem termination, and *EScaAG2* is more important for stamen formation. Expression of the three *DEF/AP3*-like genes shows organ-level differences, such that *EScaDEF3* is most strongly expressed in petals, as is *EScaDEF2*, but the latter to a lesser extent, and *EScaDEF1* is hardly expressed in petals and stamens (Yellina et al., 2010; Lange et al., 2013). In terms of trimeric protein interactions, *EScaAG1* participates in complexes of floral homeotic proteins including BBC and BCE class proteins, but *EScaAG2* does not, suggesting that the floral homeotic C function may be carried out by *EScaAG1*. Further, EScaAG2 can form homodimers, while EScaAG1 cannot, suggesting novel, yet unknown functions for *EScaAG2*. It is also notable that EScaDEF1, the E-function protein Ec-SEP3 and EScaAG1 complexes form, as well as EScaDEF2/Ec-SEP3/EScaAG1, but not EScaDEF3/Ec-SEP3/EScaAG1 (Lange et al., 2013), suggesting subfunctionalization on the level of protein interactions for these floral homeotic proteins.

The regulation of California poppy floral homeotic B and C genes differs from that of *A. thaliana*: the *AG* orthologs are activated by the *AP3* and *PI* orthologs while the *AG* orthologs repress *AP3* and *PI* activity in the carpels, as shown using VIGS experiments and by analysis of the B mutant *sei-1* (Yellina et al., 2010; Lange et al., 2013).

California poppy also served to show that the function of *INAPERTURATE POLLEN (INP1)*, a gene required for aperture formation in pollen grains to allow pollen grain germination, is conserved throughout monocots and dicots, even though sequence divergence of orthologs is comparatively high (Mazuecos-Aguilera et al., 2021).

Like in *Arabidopsis*, the *SHOOT MERISTEMLESS* (*STM*) homologs of California poppy are required for floral meristem activity (Scofield et al., 2007) and are, in combination with California poppy *CRABS CLAW* (*CRC*) orthologs required for floral meristem maintenance and timely termination. As the carpels are the last organs to be formed in the flower, their presence and number critically depends on floral meristem activity, and when the poppy *STM* genes are silenced, the floral meristem terminates prematurely leading to failure of carpel formation, as observed in *A. thaliana* (Scofield et al., 2007; Stammler et al., 2013). Conversely, more carpels are produced when the California poppy *CRC* ortholog transcription is silenced. This suggests antagonistic functions in floral meristem maintenance for the poppy *STM* and *CRC* genes (Orashakova et al., 2009; Stammler et al., 2013). *A. thaliana crc* mutants show only rarely more carpels, but the gynoecia often fail to fuse at the apex and lack nectaries. Recent work has shown that *CRC* acts as a repressing transcription factor in floral meristem termination and as a transcriptional activator in carpel fusion and nectary development (Bowman and Smyth, 1999; Groß et al., 2018). Also, in the *Phalaenopsis equestris* orchid, *CRC* homologs regulate reproductive development, specifically in the gynostemium, an organ consisting of stamens fused to the gynoecium, suggesting that the regulation of gynoecium development by *CRC* homologs is conserved between monocots and dicots (Chen et al., 2021).

*FRUITFUL* (*FUL*) and *NGATHA* (*NGA*) orthologs of poppy were shown to be involved in carpel and fruit development, indicative of deeply conserved gene functions between California poppy genes and their orthologs from *Arabidopsis*. *EcFUL1, EcFUL2* down regulated by VIGS results in shorter fruits that open prematurely and occurrence of leaf-like sepals, suggesting that sepal organ identity is compromised and the lignin deposition pattern in fruits is disturbed (Pabón-Mora et al., 2012). Lignin deposition is also disturbed in the *A. thaliana ful* mutant, resulting in a failure to form a dehiscence zone leading to fruits that rupture at random positions (Ferrandiz et al., 2000). Outside the eudicots, *FUL*-like genes are not involved in fruit dehiscence, for example, *WAP1* from wheat is required for vernalization and phase transition (Murai et al., 2003), suggesting that the involvement of *FUL*-like genes in regulation of lignification pattern for dehiscence zone formation is restricted to eudicots.

For *NGA*-like genes, California poppy VIGS-treated plants provide the only functional data outside the core eudicots, as information on mutants in grasses is lacking so far. Within eudicots, the *NGA* orthologs of *A. thaliana*, tobacco and California poppy all share that they are required for style and stigma tissue specification (Fourquin and Ferrándiz, 2014).

*CYCLOIDEA/TEOSINTE BRANCHED1*-like (*CYL/TB1*) genes of California poppy regulate plant stature, such that down regulation by VIGS enhances axillary branching, a function conserved throughout dicots and monocots. Further, the *CYL/TB1*-like genes in California poppy regulate stamen number and petal size. However, the link between stamen number regulation and *CYL/TB1*-like genes does not seem to be special to Papaveraceae, but floral organ size regulation of *CYL/TB1*-like genes is in line with a conserved function of these genes in the repeated establishment of zygomorphy within the pentapetalae. In the Papaveraceae, *CYL/TB1*-like genes do not establish zygomorphy but may regulate the extent of morphological differences between the floral organs by controlling growth (Zhao et al., 2018).

These studies show that knowledge about the conservation of developmental regulator’s gene functions across dicots can be garnered by studying California poppy as genetically tractable representative of the sister lineage to the core eudicots, and the conservation of function between monocots and dicots can be inferred by incorporating California poppy mutants or VIGS-treated plants.

### 2.2 Floral pigments

California poppy flowers contain unique carotenoids, such as eschscholtzxanthin and retro-carotene-triol (Maoka et al., 2000), which show intense yellow to orange petal pigmentation of this ornamental flower. Whereas orange flowers are more popular, color variations from white to yellow and orange are known. Barrell et al. (2010) analyzed flower color inheritance in diverse variants and showed that all white and yellow variants showed the multiple effects and total of five complementation groups were identified as single recessive loci. Interestingly, all mutations influence both petal and pollen color, suggesting that the same gene controls petal and pollen color.

Zhou et al. (2018) further investigated the carotenoid biosynthetic pathway using a Tobacco Rattle Virus-based virus-induced-gene-silencing (VIGS) approach. VIGS of early (*PDS* and *ZDS*) and late (*βOH* and *ZEP*) biosynthetic enzymes in carotenoid pathway reduced the transcripts of the target genes in the petals without the effect on other carotenoid biosynthesis gene expressions. Silencing of *PDS*, *ZDS*, *βOH* and *ZEP* genes reduced total pigment concentration by 75-90% and altered petal color. HPLC and LC-MS measurements suggested that petal color changes were caused by substantially altered pigment profiles and quantity. More recently, Pollack et al. (2019) discovered a single deletion leading to altered splicing and C-terminal truncation of phytoene synthase (*PSY*), a key enzyme in carotenoid biosynthesis mutated in multiple white petal varieties.

Whereas the key enzyme genes for retro-carotene-triol biosynthesis are still not identified yet, some candidate genes are predicted based on the draft genome sequence and transcriptome analysis for future breeding (Sato et al. unpublished data).

## 3 Medicinal use

**Figure 2:**
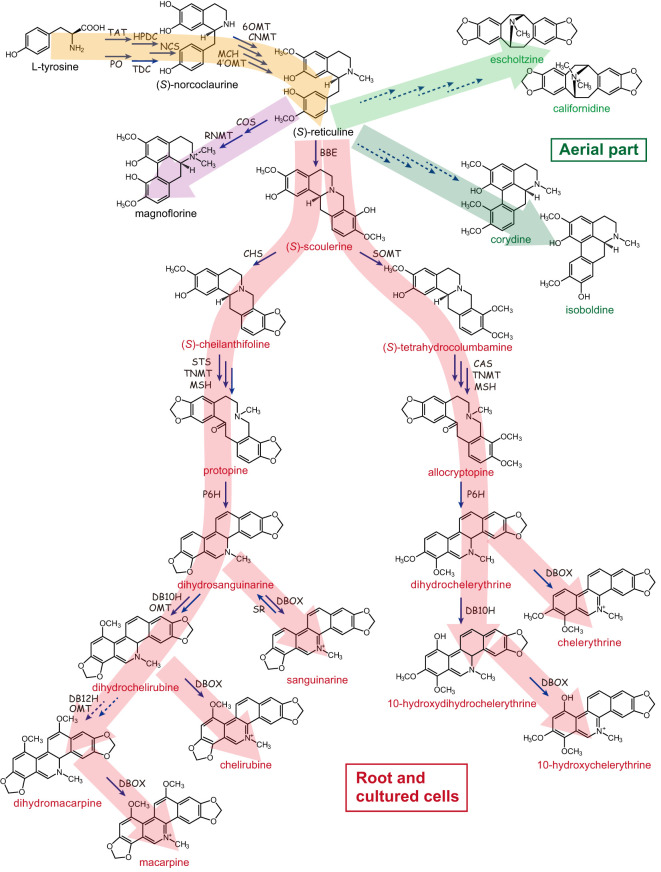
Benzylisoquinoline alkaloid biosynthetic pathway in California poppy. California poppy produces diverse array of benzylisoquinoline type alkaloids (BIAs), which include benzophenanthridine alkaloids (such as sanguinarine, chelirubine, macarpine, chelerythrine, chelilutine), protopines (protopine, allocryptopine), aporphine alkaloids (magnoflorine, corydine, isoboldine), simple benzylisoquinolines (reticuline), pavine alkaloids (californidine, escholtzine), as well as the dihydro-intermediates. The broken lines indicate that the biosynthetic enzyme-encoding genes have not been identified. TAT, tyrosine aminotransferase; HPDC, *p*-hydroxyphenylpyruvate decarboxylase; PO, phenol oxidase; TDC, tyrosine/DOPA decarboxylase; NCS, (*S*)-norcoclaurine synthase; 6OMT, (*S*)-norcoclaurine 6-*O*-methyltransferase; CNMT, (*S*)-coclaurine *N*-methyltransferase; MCH, (*S*)-*N*-methylcoclaurine 3’-hydroxylase; 4’OMT, (*S*)-3’-hydroxy-*N*-methylcoclaurine 4’-*O*-methyltransferase; COS, (*S*)-corytuberine synthase; RNMT, (*S*)-reticuline *N*-methyltransferase; BBE, berberine bridge enzyme; SOMT, (*S*)-scoulerine 9-*O*-methyltransferase; CHS, (*S*)-cheilanthifoline synthase; STS, (*S*)-stylopine synthase; CAS, (*S*)-canadine synthase; TNMT, (*S*)-tetrahydroprotoberberine *N*-methyltransferase; MSH, (*S*)-*N*-methylstylopine 14-hydroxylase; P6H, protopine 6-hydroxylase; DBOX, dihydrobenzophenanthridine alkaloid oxidase; SR, sanguinarine reductase; DB10H, dihydrobenzophenanthridine alkaloid 10-hydroxylase; DB12H, dihydrobenzophenanthridine alkaloid 12-hydroxylase. Large arrows in red denote compounds synthesized in root and cultured cells, yellow indicates starting products of BIA synthesis, and green indicates alkaloids found in aerial part.

California poppy was highly prized by Native Americans for its medicinal value based on its specialized metabolite biosynthesis, with the most prominent class being the benzylisoquinoline alkaloids (BIAs). Phytochemical analysis revealed that aerial parts and roots accumulate alkaloids, with roots showing a higher concentration of up to 1.6% alkaloids of the dry weight. These alkaloids are mainly BIAs and include benzophenanthridine alkaloids (such as sanguinarine, chelirubine, macarpine, chelerythrine, chelilutine), protopines (protopine, allocryptopine), aporphine alkaloids (magnoflorine, corydine, isoboldine, *N*-methyllaurotetanine), simple benzylisoquinolines (reticuline), pavine alkaloids (californidine, caryachine, escholtzine), as well as the dihydro-intermediates (**Figure 2**). Roots contain mainly benzophenanthridine alkaloids and protopines, whereas aerial parts are especially rich in pavine and some aporphine alkaloids (Liscombe et al., 2009; Fedurco et al., 2015; Hori et al., 2018).

Pharmacological studies of these BIAs revealed their antifungal, analgesic, anxiolytic, sedative activities (Al-Snafi, 2017). For example, extracts prepared from aerial parts of California poppy as herbal supplement show analgesic, anxiolytic and sedative effects. Rolland et al. (1991) reported that the aqueous extract reduced the behavioral performance in mice with regard to novelty preference, locomotion and rearing in two compartments parameters measured (in a familiar environment test). Further, the California poppy extract treated mice underperformed in the staircase test (non-familiar environment tests). These findings support the traditional use of California poppy herbal extract to induce sleep due to sedative properties. Fedurco et al. (2015) further assigned the depressant properties of aerial California poppy parts to chloride-current modulation at the alpha(3)beta(2)gamma(2) and alpha(5)beta(2)gamma(2) GABA(A) receptors by (*S*)-reticuline, a minor alkaloid in the herbal extract,

The biological activities of sanguinarine, the main alkaloid in California poppy root and cell cultures, have been investigated in depth and were recently reviewed by Laines-Hidalgo et al. (2022). In brief, sanguinarine shows herbivore deterrent activity as well as antimicrobial effects (Schmeller et al., 1997). These effects may be related to its cytotoxic activity, such as intercalation in nucleic acids, and inhibition of DNA and RNA synthesis. Moreover, sanguinarine can bind to negatively charged membrane surfaces and proteins as heteroaromatic iminium cation (Han et al., 2016) and it inhibits choline acetyltransferase activity in arthropods and vertebrates.

Sanguinarine, chelerythrine, and other BIAs also show antibacterial and antifungal activities (Zhao et al., 2019), whereas their direct effects on these pathogens was not analyzed *in planta*. Interestingly, antiplaque mouth washes and toothpaste containing sanguinarine were once commercially available, but were later removed from supermarket shelves due to their dramatic side effect: leukoplakia, a pre-malignant condition, of the maxillary vestibule had occurred in some consumers (Laines-Hidalgo et al., 2022).

It is further important to notice that some plant-derived alkaloids, such as protopine and (+)-*N*-methyllaurotetanine reduced the human cardiac ether-a-go-go-related gene (hERG) expression and poses a potential risk for human hERG toxicity (Schramm et al., 2014).

### 3.1 Alkaloids

The BIA pathway in California poppy provides a convenient system to study the regulation of biosynthesis and the physiological roles of BIAs (**Figure 2**). And while *Coptis japonica* (https://www.sciencedirect.com/topics/biochemistry-genetics-and-molecular-biology/coptis), *Thalictrum thalictroides* (https://www.sciencedirect.com/topics/pharmacology-toxicology-and-pharmaceutical-science/thalictrum), and *Papaver somniferum* (https://www.sciencedirect.com/topics/biochemistry-genetics-and-molecular-biology/papaver-somniferum) are also used to study BIAs, California poppy provides practical advantages. The plants are easy and permit-free to cultivate, efficient methods for cell cultures and genetic transformations are available as discussed below (Hagel and Facchini, 2013; Sato, 2013; Lotz et al., 2022).

HPLC or LC-MS analyses easily reveal alkaloid composition of California poppy (Liscombe et al., 2009; Hori et al., 2018), whereas the more traditional TLC method is also still in use (Balažová et al., 2020). Kukula-Koch (2017) reported an optimized method for the LC-ESI-Q-TOF-MS analysis for the extracts of Papaveraceae and Berberidaceae families (genera: *Papaver, Argemone, Eschscholzia, Chelidonium, Glaucium*, and *Berberis*), providing even more sensitive and precise method for BIA characterization. Based on the identification of alkaloid chemical structures and tracer experiments, the major alkaloid biosynthesis pathways and biosynthetic enzymes of BIAs have been characterized at the molecular level (Hagel and Facchini, 2013; Sato, 2013; Sato, 2020)

BIA biosynthesis commences with the conversion of tyrosine to both dopamine and 4-hydroxyphenylacetaldehyde (4HPAA) by tyrosine/dopa decarboxylase (TDC), or 4-hydroxyphenylpyruvate decarboxylase (HPDC), phenol oxidase (PO), and tyrosine amino transferase (TAT) (**Figure 2**, brown pathway). Dopamine and 4HPAA are condensed by norcoclaurine synthase (NCS) and yield (*S*)-norcoclaurine (Samanani and Facchini, 2001). (*S*)-Norcoclaurine is sequentially converted to (*S*)-reticuline by norcoclaurine 6-*O*-methyltransferase (6OMT) (Inui et al., 2007), coclaurine *N*-methyltransferase (CNMT), *N*-methylcoclaurine hydroxylase (CYP80B1; MCH) (Pauli and Kutchan, 1998), and 3’-hydroxy *N*-methylcoclaurine 4’-*O*-methyltransferase (4’OMT) (Inui et al., 2007).

(*S*)-Reticuline is converted to (*S*)-scoulerine by berberine bridge enzyme (BBE), then benzophenanthridine alkaloids (e.g., sanguinarine and macarpine) (**Figure 2**, red pathway in root and cultured cells) (Dittrich and Kutchan, 1991; Fujii et al., 2007; Ikezawa et al., 2007, 2009; Liscombe et al., 2009; Hagel et al., 2012; Beaudoin and Facchini, 2013; Takemura et al., 2013; Purwanto et al., 2017). Two enzymes unique to California poppy and identified based on draft genome sequence mining are dihydrobenzophenathridine hydroxylase (DB10H) and OMT required for dihydrochelirubine biosynthesis (Hori et al., 2018). Importantly, California poppy cell cultures can self-detoxify exogenously added benzophenanthridines by sanguinarine reductase (SR; Vogel et al., 2010). As shown in **Figure 2**, the biosynthetic pathway of sanguinarine and related chelerythrine and chelirubine in underground parts and cultured cells of California poppy has been almost completely elucidated.

The characterization of BIA biosynthetic enzymes was the prerequisite to elucidate the mechanisms of their transcriptional regulation (Yamada and Sato, 2021). Firstly, *CjWRKY1* and *CjbHLH1* were identified as comprehensive transcriptional activators of biosynthetic enzyme genes in BIA biosynthesis of *C. japonica* cells (Kato et al., 2007; Yamada et al., 2011a). Later, their homologs were isolated from California poppy (*Ec*WRKY and *Ec*bHLH1-1/1-2) allowing for the characterization of the regulation of BIA biosynthesis in California poppy (Yamada et al., 2015; Yamada et al., 2021b). Interestingly, *Cj*bHLH1 and *Ec*bHLH1-1/1-2 are non-MYC2-type bHLH transcription factors and their homologs are only found in BIA-producing plant species (Yamada et al., 2011b). Whereas WRKYs were also identified in California poppy genome, the heterologous expression of *Cj*WRKY1 in California poppy showed limited activation of BIA biosynthetic enzyme genes and only partial increase in BIA production, suggesting that their function is not fully interchangeable (Yamada et al., 2017). Detailed genome-wide analysis of the California poppy WRKY transcription factor family combined with transcriptome analysis suggested that this gene family is involved in the regulation of BIA biosynthesis and is possibly also associated with the accumulation and translocation of BIAs in California poppy (Yamada et al., 2021b).

Further transcriptome analysis and genome mining revealed several transcription factor genes that are strongly upregulated in response to methyl jasmonate (MeJA), such as *EcAP2/ERF2*, *EcAP2/ERF3* and *EcAP2/ERF4* (Yamada et al., 2020). MeJA also sequentially induced the expression of *bHLH* and *WRKY* genes as well as of BIA biosynthetic enzyme and transporter genes (Yamada and Sato, 2021). This information on additional BIA regulatory transcription factors and MeJA as inducing phytohormone, all obtained in California poppy, can be highly useful to dissect the regulation of BIA biosynthesis in diverse plant species.

Unfortunately, molecular information of the genes encoding the biosynthetic enzymes for pavine-type BIAs in the aerial parts of California poppy, such as californidine and escholtzine (**Figure 2**, green pathway) is still missing (Hori et al., 2018). Moreover, while pavine-type alkaloids are most prominent in leaves, their physiological role is unknown. And, the important question on why pavine-type alkaloids are found in leaves and benzophenanthridine alkaloids accumulate in roots and how this relates to the defense against different pathogens would be interesting to study in California poppy.

We also note that California poppy has limitations, due to the lack of some commercially important BIAs, such as noscapine and morphinan alkaloids (Hagel and Facchini, 2013; Sato, 2020). The biosynthesis of morphinan alkaloids in opium poppy requires cell-type-specific localization of the biosynthetic enzymes. *In situ* localization of their transcripts indicated that seven biosynthetic enzymes (6OMT, CNMT, CYP80B, 4’OMT and BBE involved in reticuline biosynthesis, and SAT and COR in the morphine pathway) were localized in sieve elements, whereas proteins were localized in the supporting companion cells, demonstrating a complex spatial organization of morphinan alkaloids (Lee et al., 2013; Ozber et al., 2022). California poppy could serve as preferred host system to reconstruct the morphinan pathway including genes encoding for the biosynthetic enzymes, translocators, and transcription factors genes involved in cell differentiation. This would be a prime example for synthetic biology to recreate a highly complex biosynthetic pathway of a commercially extremely valuable pharmaceutical.

Undoubtedly, the California poppy draft genome provides a useful platform to study the evolution of BIA biosynthesis and its regulation. Phylogenomic approaches using whole genome sequences of five benzylisoquinoline alkaloid (BIA)-producing species from the Ranunculales and Proteales orders including California poppy revealed the sequence and timing of evolutionary events leading to the diversification of BIA alkaloids (Li et al., 2020). 1-Benzylisoquinoline is a pivotal intermediate in the synthesis of many BIAs and phylogenomic analyses revealed parallel evolution in the orders of Ranunculales and Proteales, which diverged -122 million years ago (MYA), with the Ranunculales producing (*S*)-reticuline and the Proteales the related (R/S)-norcoclaurine. Berberine is present in species across the Ranunculales but lacking in Proteales, and homologs of genes essential for the protoberberine class production were found throughout the Ranunculales. However, benzophenanthridine class is specific to the Papaveraceae family within Ranunculales (**Figure 1**), and its biosynthetic genes emerged after the Papaveraceae separated from the other Ranunculales, around 110 MYA. Their origin also predates the split of the three Papaveraceae species (opium poppy, California poppy, and *Macleaya cordata*) at approximately 77 MYA. The phthalideisoquinoline noscapine and morphinan classes of BIAs are exclusive to the opium poppy lineage (Li et al., 2020). In addition, predicted protein-encoding genes and comparative analysis using genome sequences of BIA-producing plants, opium poppy and *Aquilegia coerulea*, showed many additional candidate genes encoding for biosynthetic enzymes, transcription factors, and transporter genes involved in the BIA pathway (Yamada et al., 2021a).

## 4 Genetic resources

The species California poppy shows a stunning variation in floral and vegetative traits observed in natural populations and its adaptability suggests high genetic variation. Cytological observations and classical genetic experiments by Ernst and Cook (Ernst, 1958; Cook, 1962) revealed that California poppy has 6 chromosomes, and is self-incompatible to the largest extent, even in the naturalized populations, and genetic barriers to outcrossing were not identified so far. The powdery nature of the pollen allows also for wind pollination, but the flowers are mainly pollinated by insects. While the large flowers do not exudate nectar, they abundantly provide pollen. Beetles, bees and bumblebees contribute significantly to California poppy pollination, but also thrips and hover flies were observed to visit the flowers (Cook, 1962). The open breeding system of this species allows novel adaptive trait combinations and permits differentially adapted population existing close to each other (Cook, 1962) and may be a prerequisite of its invasiveness.

### 4.1 Sequence resources

Several genetic resources have been developed to facilitate California poppy research with the advantages of relatively small genome size (503.8 Mb): 14 microsatellite markers are available for population genetic analysis that have been utilized to characterize the highly invasive populations in Chile (Véliz et al., 2012). And EST database has been established to facilitate gene discovery (Carlson et al., 2006), and the sequence information garnered from the ESTs was used to generate microarrays for differential gene expression studies (Zahn et al., 2010). In addition to these transcriptomes, RNAseq data for different stages of carpel development obtained by laser microdissection are available from California poppy (Kivivirta et al., 2019).

Meanwhile, a draft genome sequence was published (Hori et al., 2018; Eschscholzia Genome DataBase, http://eschscholzia.kazusa.or.jp) and a reference-quality genome sequencing effort in combination with a transcriptome atlas is about to be completed by the Open Green Genome initiative with the data being deposited in the phytozome database (OGG, https://phytozome-next.jgi.doe.gov/ogg/) for easy access. Further, we are generating a large number of high quality transcriptome data for diverse tissues and these will be available for the research community *via* a web-based multi-omics platform. This will allow *in silico* analysis of the genome and digital gene expression analysis along the entire transcriptome and be useful for gene network constructions.

### 4.2 Resources for genetic manipulation

In addition to sequence information, questions regarding the conservation of gene functions can be addressed by knocking down gene expression of target genes by Virus-Induced Gene Silencing (VIGS). This method allows gene function analysis in a simple and time efficient way by manipulating the plant’s immune reaction towards RNA viruses such that the expression of endogenous genes is reduced (Wege et al., 2007; Rössner et al., 2022). This method was employed several times to elucidate the function of transcription factors involved in vegetative and reproductive development (Orashakova et al., 2009; Yellina et al., 2010; Lange et al., 2013; Stammler et al., 2013; Zhao et al., 2018). While the effect of VIGS is transient and cannot be transferred to subsequent generations, a second, reliable method for stable transformation and regeneration of mature plants is now available (Park and Facchini, 2000; Lotz et al., 2022). Here, *Agrobacterium tumefaciens*-mediated transformation of cotyledons and subsequent regeneration is works efficiently but is time-consuming in California poppy, requiring at least eight months from the transformation event to mature plants (Park and Facchini, 2000; Lotz et al., 2022).

Whereas prolonged modulation of gene expression with *A. tumefaciens* mediated stable transformation, or VIGS/virus-based expression is useful for the functional characterization of biosynthetic enzyme or developmental genes, transient assays using protoplasts are faster to characterize certain gene functions such as transcription factor genes regulating BIA biosynthesis. Protoplasts are prepared from plant cells after the digestion of cell walls with cellulase, pectinase and other cell wall digesting enzymes. Protoplasts take up DNA, RNA, or proteins easily when treated with polyethylene glycol (PEG), or electric stimulus. For example, the efficacy of double-stranded (ds) RNAs prepared against candidate transcription factor encoding genes or over-expression plasmids for transcription factor encoding genes were examined in protoplasts of *C. japonica*, a relevant model for BIA-producing plants using PEG-mediated transformation. Suppression effects of TFs on biosynthetic enzyme genes were successfully monitored by quantitative reverse transcription (RT)-polymerase chain reaction (PCR) in *C. japonica* (Dubouzet et al., 2005). Since mesophyll protoplasts are rather easily isolated from California poppy leaves (data not shown), similar system will be applicable, for example to characterize the pavine-biosynthesis in leaf tissue.

### 4.3 Cell culture systems

California poppy cell cultures have been intensively used to study BIA pathway, since cell cultures produce the major BIAs and provides sufficient materials for biochemical and molecular genetics characterization (Sato, 2013). Cell culture systems are also useful to modify biosynthetic pathways using genetically transformed cultures (Sato et al., 2001; Fujii et al., 2007; Inui et al., 2007), and to test the effect of chemicals such as elicitors from pathogens or MeJA as BIA pathway activator (Tanahashi and Zenk, 1990; Färber et al., 2003; Ikezawa et al., 2009).

In fact, introduction of the *C. japonica* scoulerine 9-*O*-methyltransferase (*Cj*SMT) gene into BIA biosynthesis in a California poppy cell culture system shifted the metabolic flow from the sanguinarine type to chelerythrine type (Sato et al., 2001, **Figure 2**). Whereas both introduced *Cj*SMT and endogenous cheilanthifoline synthase (*Ec*CYP719A2/A3) accept scoulerine as substrate, the highly reactive *Cj*SMT dominated the pathway when compared to the endogenous *Ec*CYP719A2/A3. Similarly, when the *C. japonica* (*S*)-tetrahydroberberine oxidase (*Cj*THBO) was introduced to California poppy cells, *Cj*THBO hijacked the intermediates in benzophenanthridine biosynthesis to convert them into protoberberine type products (Matsushima et al., 2012).

The physiological role of down-regulation using antisense RNA, co-suppression or gene-knockout with CRISPR/Cas 9 is also effectively monitored in cell culture systems. For example, the effects of RNA-interference (RNAi) of berberine bridge enzyme (BBE) gene in BIA pathway can be detected as the accumulation of the key intermediate reticuline with substantial production of 7-*O*-methylated derivative of reticuline, laudanine, which indicates the dynamics of metabolism (Fujii et al., 2007).

Sterilized California poppy seedlings grown on 1% agar medium containing Murashige–Skoog inorganic salts under continuous light (100 μE/m^2^/s) at 25°C are preferable materials for cell culture or transformation with *Agrobacterium tumefaciens.* Calli generally form after 2 months (about three successive selection cultures) on culture medium containing appropriate plant growth factors such as auxin, cytokinin, and antibiotics for selecting the transgenics. Transformation efficiency, regeneration/callus formation efficiency, and secondary metabolite productivity can vary considerably and require seed variety comparisons as preliminary experiments. For the establishment of an embryogenic culture, juvenile tissues such as shoot meristem and immature seed are often preferred materials (Takemura et al., 2010). Whereas Cauliflower Mosaic Virus 35S is a commonly used promoter sequence to over-express desired genes constitutively in host plant cells, gene expression in specific tissues or developmental stages requires carefully selected promoters and even enhancers. Thus, for alkaloid engineering, more research on the regulation of metabolic pathway and specific gene expression profiles is needed. One example for a comprehensive characterization of biosynthetic enzymes was done with the genes encoding for the two *C. japonica* enzymes norcoclaurine 6-*O* methyltransferase (*Cj*6OMT) and 3’-hydroxy-*N*-methylcoclaurine 4’-*O*-methyltransferase (*Cj*4’OMT). They were over-expressed in California poppy cell cultures and showed different effects. Over-expression of *Cj*6OMT increased the alkaloid content to 7.5 times greater than that of the wild type, whereas the over-expression of *Cj*4’OMT had only a marginal effect (Inui et al., 2007).

Cell culture systems proved also useful to dissect BIA induction and its role in the molecular mechanism of phytopathogen defense. Whereas jasmonate treatment is commonly used to activate BIA biosynthesis (Färber et al., 2003; Ikezawa et al., 2009), Balažová et al. (2020) examined salicylic acid (SA), and simultaneous or sequential treatment of SA and L-tyrosine in cell cultures to enhance production of macarpine, a BIA specific to few Papaveraceae species only. Angelova et al. (2010) used root-derived cell cultures to characterize the elicitation mechanism, initiated by a short contact to low concentrations of a yeast glycoprotein elicitor, which led to the transient acidification of the cytoplasm. In contrast to low concentration treatment, high elicitor concentration signal increased jasmonate concentration and triggered hypersensitive cell death, resulting in massive mRNA decay.

Taken together, transient and stable genetic transformation methods have been established for California poppy to interrogate gene functions in different contexts. For metabolic engineering and the analysis of metabolite biosynthesis regulation, stable genetic transformation of cell culture systems have been used extensively. For the analysis of developmental regulators, fully grown plants are required and VIGS was used efficiently to unravel their function and regulatory circuits, even though this method is transient. The novel method for stable transformation and regeneration of California poppy will provide even more possibilities, especially for targeted, heritable mutagenesis by CRISPR-Cas.

## 5 Outlook

California poppy’s high level of genetic diversity comes with the cost of being an obligate outcrossing plant. This suggests that the level of heterozygosity is high and the isogenic and even near-isogenic lines production is very challenging. However, homozygous mutants can be created by sibling crossing. Moreover, CRISPR-Cas guided genome editing introduced by *Agrobacterium*-mediated transformation and regeneration provides an efficient means to elucidate gene function in homozygous knock-out mutants. Further, VIGS can be combined with CRISPR-Cas such that the guide RNAs may be delivered by VIGS to a plant carrying a CRISPR-Cas expressing transgene. These future developments of the California poppy toolkit will enhance the potential of this already established model organism to study, for example, BIA biosynthesis and its regulation in fully grown plants and link this with developmental genetics analyses. Availability of many high quality transcriptome datasets allows the calculation of gene networks based on genes’ co-expression to identify whole modules of putatively interacting genes. This type of analysis is independent of candidate genes associated to biological processes in other species, such as *Arabidopsis*, and is thus bias-free.

Additionally to California poppy genome and transcriptome datasets being generated, these datasets are becoming available also for other Ranunculales, allowing comparative analyses. Several Ranunculales genomes have been published recently, some even at chromosome-level, including *M. cordata* (Liu et al., 2017), *Coptis chinensis* (Liu et al., 2021), *P. somniferum* (Guo et al., 2018), *Papaver rhoeas, Papaver setigerum* (Yang et al., 2021), and *A. coerulea* (Filiault et al., 2018), *Aquilegia oxysepala* (Xie et al., 2020), *Thalictrum thalictroides* (Arias et al., 2021), *Kingdonia uniflora* (Sun et al., 2020), *Akebia trifoliat*a (Huang et al., 2021), and *Corydalis tomentella* (Xu et al., 2022).

While genome sequencing requires the extraction of a single sample of high molecular weight DNA, transcriptome analysis requires the collection of several biological replicates for many tissues, stages, and/or treatments, rendering this method more time consuming and laborious. While for *Nigella damascena, A. coerule*a, and *T. thalictroides* more extensive transcriptomes datasets have already been published facilitating gene identification in Ranunculales and gene expression analysis to a limited extent (Meaders et al., 2020; Zhang et al., 2020; Arias et al., 2021). However, the datasets often comprise only few tissues and an insufficient number of replicates to allow for digital gene expression analysis. Furthermore, gene expression comparison of orthologous genes is challenging at the present state, if possible at all. Comparable datasets including representatives covering all Ranunculales subfamilies is desirable for cross-species comparison of gene expression data. Sufficient expression data for each representative species is further required for co-expressed gene network calculations and to compare these networks between species to learn about novel network nodes that may correspond to morphological or metabolic novelties in the Ranunculales.

Furthermore, California poppy allows the fusion of two formerly separated fields in plant biology: developmental genetics and regulation of metabolism: floral homeotic genes specify floral organ identity and any anomaly in the structure or expression of these genes apparently may result in morphological variations of the flower/capsule and consequently in the alkaloid yield. One such recessive mutation, *aco* (*androcarpel organ*), has been described in the opium poppy (Prajapati, 2002), in which androcarpels are formed in place of stamens in the mutant flowers. The androcarpel walls synthesized and accumulated alkaloids similar to the main carpel walls and thus provided a means for increasing the carpel wall husk mass and alkaloid yield. Singh et al. (2017) also reported the presence of major BIAs in the carpeloid stamens of the floral homeotic mutant *OM*, unlike in wild type stamens, indicating functional similarities between the carpeloid stamen and the capsule wall in their capacity to synthesize BIAs. Whereas California poppy does not synthesize morphinan alkaloids, it also accumulates BIAs in floral organs and thus allows the study of the interplay of regulatory genes in flower morphogenesis and specialized metabolism, i.e., carotenoid and BIA biosynthesis for crop improvement in future breeding programs of Papaveraceae species.

## Author contributions

AB, YY, and FS wrote the draft and final version of the manuscript. All authors contributed to the article and approved the submitted version.
